# Down-converted photon pairs in a high-*Q* silicon nitride microresonator

**DOI:** 10.1038/s41586-025-08662-3

**Published:** 2025-03-19

**Authors:** Bohan Li, Zhiquan Yuan, James Williams, Warren Jin, Adrian Beckert, Tian Xie, Joel Guo, Avi Feshali, Mario Paniccia, Andrei Faraon, John Bowers, Alireza Marandi, Kerry Vahala

**Affiliations:** 1 T. J. Watson Laboratory of Applied Physics, California Institute of Technology, Pasadena, CA USA; 2 Department of Electrical Engineering, California Institute of Technology, Pasadena, CA USA; 3 Anello Photonics, Santa Clara, CA USA; 4 ECE Department, University of California Santa Barbara, Santa Barbara, CA USA

**Keywords:** Microresonators, Single photons and quantum effects, Nonlinear optics, Integrated optics

## Abstract

Entangled photon pairs from spontaneous parametric down-conversion (SPDC)^1^ are central to many quantum applications^2–6^. SPDC is typically performed in non-centrosymmetric systems^7^ with an inherent second-order nonlinearity (*χ*^(2)^)^8–10^. We demonstrate strong narrowband SPDC with an on-chip rate of 0.8 million pairs per second in Si_3_N_4_. Si_3_N_4_ is the pre-eminent material for photonic integration and also exhibits the lowest waveguide loss (which is essential for integrated quantum circuits). However, being amorphous, silicon nitride lacks an intrinsic *χ*^(2)^, which limits its role in photonic quantum devices. We enabled SPDC in Si_3_N_4_ by combining strong light-field enhancement inside a high optical *Q*-factor microcavity with an optically induced space-charge field. We present narrowband photon pairs with a high spectral brightness. The quantum nature of the down-converted photon pairs is verified through coincidence measurements. This light source, based on Si_3_N_4_ integrated photonics technology, unlocks new avenues for quantum systems on a chip.

## Abstract (web summary)

Spontaneous parametric down-conversion was used to generate narrowband photon pairs with a high spectral brightness in a high-*Q* silicon nitride microresonator.

## Main

Since its demonstration^1^, spontaneous parametric down-conversion (SPDC) has been widely used to generate entangled photon pairs for a range of applications^2–6^. Traditionally, non-centrosymmetric^7^ materials with a strong second-order nonlinearity *χ*^(2)^ have been used for SPDC. The advent of thin films of non-centrosymmetric materials has made SPDC possible in integrated photonics. For example, on-chip waveguides in lithium niobate^8,9^ and aluminium nitride^10^ enable quantum light sources at smaller footprints, and their fabrication uses scalable methods. Alongside non-centrosymmetric systems, it has also been possible to induce an effective *χ*^(2)^ in amorphous systems with the photogalvanic effect. Historically, this approach was first demonstrated in optical fibres^11^ where second-harmonic generation (SHG) was observed. More recently, a photogalvanic-induced *χ*^(2)^ has also been observed in Si_3_N_4_ waveguides and used to generate efficient SHG^12–16^. Also, SPDC at low photon flux rates has been observed in Si_3_N_4_ waveguides^17^.

The relatively weak nature of the photogalvanic-induced *χ*^(2)^, compared to non-centrosymmetric systems, poses a challenge for its application to SPDC. Nonetheless, if overcome, SPDC in the Si_3_N_4_ system would offer an unparalleled capability for photonic integration, including the heterogeneous integration with active components^18,19^. Moreover, the recent development of Si_3_N_4_ waveguides with an ultra-low optical loss^20,21^ makes this platform ideally suited for both the transport of quantum states on-chip and the seamless (that is, nearly lossless) coupling of these waveguides to an efficient SPDC source.

To overcome the weak second-order nonlinearity, we implemented SPDC in high quality factor (high-*Q*) Si_3_N_4_ resonators. We achieved pair generation rates of up to 0.8 million pairs per second on-chip at a high spectral brightness. Furthermore, the associated resonance linewidths ensured that the SPDC was narrowband, so that the photon-pair source could be applied in applications such as quantum memory^4,22^ and entanglement swapping^23,24^ without the need for a lossy spectral filter. Significantly, this generation rate was attained with an on-chip pump power of only 1.5 mW, a level that is readily attainable using semiconductor lasers heterogeneously integrated to the Si_3_N_4_ SPDC device^19^. To confirm the quantum nature of these down-converted photons, we performed coincidence measurements, finding a coincidence-to-accidental ratio ranging from 50 to 2,500 depending upon the on-chip pump power. We also describe a transient behaviour in the SPDC process, which requires a periodic refresh of the photogalvanic effect.

## SPDC in silicon nitride

**Fig. 1:**
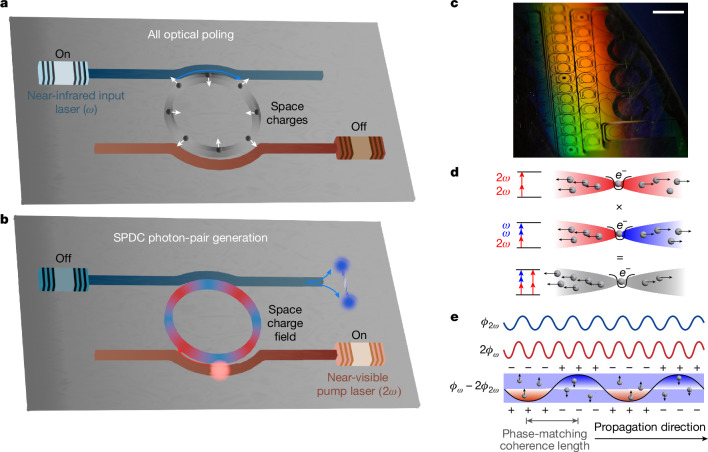
Principle of photogalvanic-induced SPDC. **a**, Depiction of the formation of a periodic space-charge grating in a Si_3_N_4_ microresonator. Input light at *ω* (1,560 nm) is coupled into the resonator, and an initial weak second-harmonic signal at 2*ω* (780 nm) is generated through symmetry breaking, for example, at the waveguide–cladding interface^33^. The co-propagating input light and second-harmonic signal induce a periodic space-charge distribution. The resulting electric field combined with the inherent *χ*^(3)^ of Si_3_N_4_ creates an effective *χ*^(2)^, thereby further enhancing the second-harmonic signal. **b**, Depiction of SPDC in a Si_3_N_4_ microresonator. After the space-charge grating forms (**a**), pump light at 780 nm is coupled into the resonator and near-infrared entangled photon pairs are generated by SPDC at 1,560 nm. The lasers (both 1,560 and 780 nm) and resonators can be integrated on a semiconductor photonic chip. **c**, Photograph of a section of the 8-inch wafer showing the many Si_3_N_4_ resonators used in this work (highlighted region). **d**, The photogalvanic process relies upon two-photon transitions (780 nm) and three-photon transitions (one 780 nm photon and two 1,560 nm photons) that occur simultaneously in Si_3_N_4_. Quantum interference^34^ of these two processes breaks the symmetry and creates a field that induces drifting by the conduction electrons generated by the absorption process. **e**, Diagram of the charge distribution with respect to the phase of two optical signals along the propagation direction. The net space-charge accumulation is proportional to the phase difference between two optical frequencies (*ϕ*_2*ω*_ − 2*ϕ*_*ω*_). This space-charge distribution and the resulting electric field quasi-phase-match the momentum difference for SHG. Scale bar, 5 mm.

Figure 1a,b illustrates the preparation and application of the SPDC process in a high-*Q* Si_3_N_4_ microresonator with an effective *χ*^(2)^. First, 780 nm (near-visible) pump photons enter the microresonator and interact with a spatially varying *χ*^(2)^ to produce 1,560 nm (near-infrared) photon pairs through SPDC. The pump and SPDC photons are resonant in the cavity, and the resulting resonant enhancement makes it possible to leverage the relatively weak *χ*^(2)^ to realize significant photon-pair generation rates. Moreover, the process is quasi-phase-matched, but unlike conventional quasi-phase-matching in which lithographically defined electrodes are required to write domains through the application of an external electric field, quasi-phase-matching was performed without lithography through an all-optical induction process referred to as all-optical poling^13,15,25^. This process writes a periodic space-charge field through the photogalvanic effect, and this field in combination with the third-order Kerr effect (*χ*^(3)^ nonlinearity) induces an effective *χ*^(2)^ that reverses sign periodically to quasi-phase-match the SPDC process. For a high-*Q* resonator, it is only necessary that the pump and subharmonic resonances are frequency aligned by tuning the temperature of the resonator. Figure 1a illustrates the resulting optically induced *χ*^(2)^ inside a silicon nitride microresonator. Further details of the photogalvanic process are provided in the caption of Fig. 1a–e.

## SPDC photon spectra

**Fig. 2:**
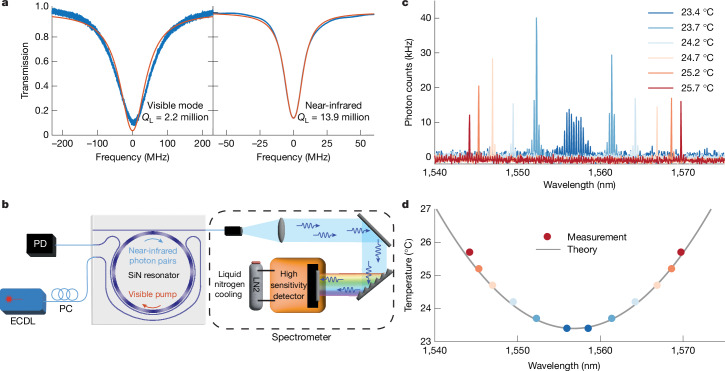
Experimental results for photon-pair generation. **a**, Measurements of resonator *Q* factors for the near-visible (left) and near-infrared (right) modes. Blue traces are measurements, and red traces are the theoretical fit. *Q*_L_ stands for loaded *Q* factor. **b**, Experimental set-up for SPDC spectral measurements. A 780 nm external-cavity diode laser pumps the resonator, and SPDC photons are analysed by a liquid-nitrogen-cooled high-sensitivity spectrometer. **c**, SPDC photon spectra measured at several chip temperatures as given in the legend. **d**, Measured wavelength-temperature dependence (dots) is plotted along with the theoretical calculation based on thermal frequency tuning. ECDL, external-cavity diode laser; PC, polarization controller; PD, photodetector; LN2, liquid nitrogen.

The resonator uses Si_3_N_4_ waveguides cladded with silica. The waveguide core (5 μm wide and 0.1 μm thick) was multimode. This wider dimension reduced the loss and boosted the *Q* factor. The free spectral range (FSR) of the resonator was 35 GHz. Two different waveguide-resonator couplers were used to couple 1,560 and 780 nm light (Fig. 1a–c). The pumping and fundamental coupler waveguides had designs with slightly different geometries to ensure coupling to the fundamental mode of the resonator. The *Q* factors of the near-infrared mode and the near-visible mode were retrieved by fitting the linewidth of the Lorentzian transmission when a laser was scanned across the resonance of the cavity. The measured *Q* factors are shown in Fig. 2a. After establishing an operating temperature at which frequency-matching occurs between the near-visible and near-infrared modes, the on-chip near-infrared pumping power of 60 mW produced a second-harmonic signal through the build-up of the photogalvanic *χ*^(2)^. The concomitant SHG achieved an efficiency of 650% per watt. Details of the set-up and characterization of the SHG are included in the Methods.

Once the effective *χ*^(2)^ was created, the resonator was pumped at the near-visible mode to generate SPDC using a continuous-wave laser. The down-conversion signal was collected through the 1,560 nm port. Beneficially, the 1,560 nm coupler also served as a filter that suppressed coupling of the 780 nm pump light with a high extinction ratio of 60 dB. The spectral distribution of the SPDC light was measured using a high-sensitivity spectrometer (Fig. 2b). The results are shown in Fig. 2c. The measured spectra were integrated over 10 s, and the indicated photon count rate is on-chip.

The down-conversion process can be either degenerate or non-degenerate depending on the frequency-matching condition, which is controlled by the chip temperature. Degenerate down-conversion happened when the near-visible mode frequency was exactly twice the frequency of the near-infrared mode, the same condition as for SHG. By increasing the temperature away from the second-harmonic matching frequency, the near-visible mode frequency mismatched the central near-infrared mode (data in Fig. 2c), resulting in down-conversion to non-degenerate modes. However, because of the second-order dispersion, the down-conversion process experienced a gradual frequency mismatch with increasing amounts of non-degeneracy (the signal and idler modes become more spectrally separated). This frequency mismatch is given by *ω*_*m*_ + *ω*_−*m*_ − 2*ω*_0_ = *m*^2^*D*_2_, where *ω*_0_ is the near-infrared frequency of perfect matching with the pump frequency, and *ω*_*m*_ and *ω*_−*m*_ are the frequencies of the signal and idler modes, with *m* a relative mode number such that *ω*_0_ corresponds to *m* = 0. Also, *D*_2_ is the second-order dispersion coefficient. The spectral locations of the signal and idler waves versus temperature are plotted in Fig. 2d. A model (black curve in Fig. 2d) based on frequency tuning versus temperature measurements and measurements of the second-order dispersion agrees well with this data. Details of the model and measurement are given in the Methods.

The continuous-wave pump and the narrow linewidth of the resonator ensured that the signal and idler photons were spectrally separable, provided that the phase-matching restricted emission into a single idler and single signal mode resonant frequency. This occurred for the two cases presented in Fig. 2c (orange and red lines for temperatures of 25.2 and 25.7 °C). As discussed in the Methods, the resonator second-order dispersion as well as the resonator linewidth (or, correspondingly, the optical *Q* factor) can be used to ensure the single idler and signal condition for spectral separability. Most notably, the use of a larger FSR resonator (over 74 GHz) would guarantee this condition for all cases. Conversely, multimode frequency-bin entangled sources can be useful for encoding information when photon pairs are generated across seveal cavity resonances (dark blue and light blue traces in Fig. 2c)^26^. Nonetheless, in the current resonator design, several simultaneous pair wavelengths can occur due to second-order dispersion.

## Quantum nature of the down-converted photons

**Fig. 3:**
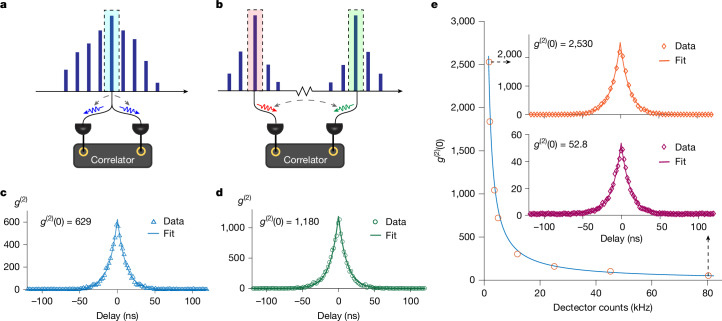
Second-order quantum-correlation measurement. **a**,**b**, Illustration of the measurement set-up showing degenerate (**a**) and non-degenerate (**b**) cases. **c**, Measured *g*^(2)^ for the degenerate SPDC case through self-correlation. **d**, Measured *g*^(2)^ for the non-degenerate SPDC case through cross-correlation of the signal and idler photons. **e**, Measured *g*^(2)^(0) of non-degenerate SPDC at different pump powers. The red open circles show the *g*^(2)^(0) at different detector count rates, and the blue curve is an inverse proportional fit. Insets, *g*^(2)^(0) = 2.530 was obtained with a 17.2 kHz on-chip photon-pair generation rate, and *g*^(2)^(0) = 52.8 was obtained with a 795 kHz on-chip pair generation rate.

The quantum nature of the SPDC photon pairs was verified by measuring the second-order correlation *g*^(2)^. Experimentally, down-converted photons were detected by superconducting single-photon detectors (SNSPDs) manufactured by ID Quantique. Both degenerate SPDC (Fig. 3a) and non-degenerate SPDC (Fig. 3b) were studied by adjusting the temperature of the resonator. In the degenerate case, a spectral filter with a 10-GHz bandwidth selected only the centre mode, and the filtered photon-pair stream was passed through a 50/50 coupler before being detected by the SNSPDs. In the non-degenerate case, the signal and idler photons (typically separated by tens of nanometres) were selected with two 10-GHz-bandwidth spectral filters before being detected by the SNSPDs. In both cases, the second-order correlation was measured by counting the two-photon coincidence rates at different delay times for the two optical paths.

The two cases were first studied at a relatively low pumping power of 50 μW (on-chip). Measurements of *g*^(2)^ versus the time delay for the degenerate and non-degenerate cases are presented in Fig. 3c,d, respectively. The data were fitted (blue and green curves) using the theoretical results $${g}_{\text{degenerate}}^{(2)}=1+(1/4R{\tau }_{{\rm{L}}})\exp (-\tau /{\tau }_{{\rm{L}}})$$ and $${g}_{\text{non-degenerate}}^{(2)}\,=$$$$1+(1/2R{\tau }_{{\rm{L}}})\exp (-\tau /{\tau }_{{\rm{L}}})$$ for cavity-enhanced SPDC^10,27^. In these expressions, *R* is the photon-pair generation rate, *τ*_L_ is the loaded cavity lifetime and *τ* is the delay between two detectors. The data fit gave cavity lifetimes of 11.2 and 11.9 ns for Fig. 3c,d, respectively, which are in good agreement with the lifetime of 11.5 ns obtained from the 13.9 million cavity *Q* measurement. Moreover, the fitted on-chip pair generation rates were 35 and 33 kHz, respectively. These should be compared with the measured detector count rates of 3.7 and 3.4 kHz for Fig. 3c,d, respectively. Accounting for the 9.8 dB chip to detector loss, these measurements are in good agreement with the fitting to theory.

In Fig. 3e, we examine the relation between *g*^(2)^(0) and the photon-pair generation rate *R*. A larger generation rate reduced *g*^(2)^(0) because there was a higher probability of detecting uncorrelated photons. The observed inverse relationship of *g*^(2)^(0) on generation rate is consistent with the theory. *g*^(2)^(0) values spanning from 53 to 2,530 were measured. These values correspond to detector count rates from as high as 80.2 kHz (1.5 mW on-chip pump power) to as low as 1.8 kHz (25 μW on-chip pump power). As an aside, the 80.2 kHz at-detector rate corresponds to a 795 kHz on-chip rate, which is an exceptionally strong level for a non-centrosymmetric material.

During the experiment, we observed that the down-converted photon flux rate decayed over time. We believe that this was caused by washing out of the space-charge distribution by the near-visible pump. At this wavelength, electrons may be excited to the conduction band through multiphoton absorption. These free charges could then drift to neutralize the accumulated charges and thereby reduce the effective *χ*^(2)^. At 1.5-mW on-chip pump power, the reduction of pair production for non-degenerate conditions was relatively slow, taking several minutes to impact the measurement. Moreover, the generation rate fully recovered when pumping was combined with a near-infrared signal. Specifically, this decay phenomena did not occur for SHG when near-infrared and visible light were simultaneously present. Details about this behaviour are provided in Methods.

## Discussion and outlook

**Fig. 4:**
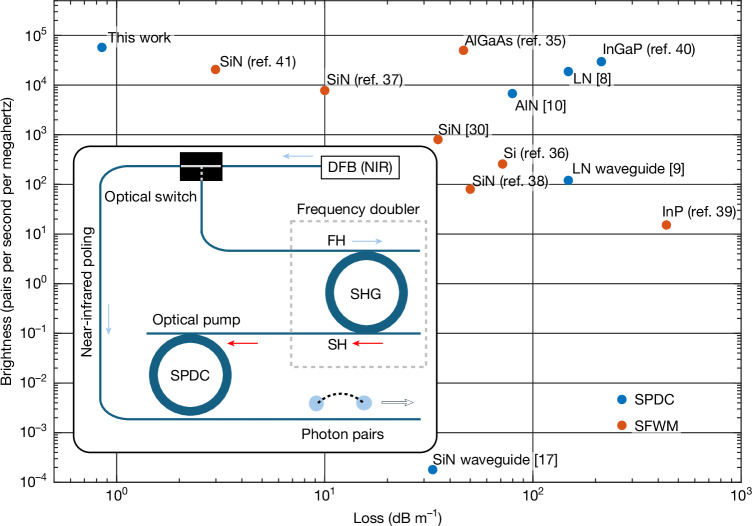
Measured SFWM and SPDC brightness and loss performance comparison. The results achieved in this work are compared with other integrated quantum photonics platforms using two metrics, waveguide optical loss and source brightness^8–10,17,30,35–41^. The source brightness metric has been frequently used in this context. The waveguide loss metric is of critical importance for integrated quantum systems as it impacts quantum state transport across the semiconductor chip. The brightness was calculated from the maximum reported pair generation rate divided by the spectral span (from the resonator total quality factor). The waveguide loss was estimated from the resonator intrinsic quality factor, if not reported. The plot shows both SFWM (red points) and SPDC (blue points) processes. The pump powers used in the references are as follows: 1.5 mW (this work), 13.4 μW (ref. ^8^), 0.45 mW (ref. ^9^), 2.3 mW (ref. ^10^), 0.43 mW (ref. ^17^), 0.45 mW (ref. ^30^), 25 μW (ref. ^35^), 60 μW (ref. ^36^), 2.0 mW (ref. ^37^), 6.2 mW (ref. ^38^), 22 μW (ref. ^39^), 1.2 μW (ref. ^40^) and 0.33 mW (ref. ^41^). Inset, conceptual figure for an integrated photonic SPDC source featuring a single integrated near-infrared (telecom) DFB pump that is frequency-doubled (SHG) by a high-*Q* Si_3_N_4_ resonator to provide the near-visible pump for a high-*Q* Si_3_N_4_ resonator SPDC source. DFB, distributed feedback laser; FH, first harmonic; NIR, near-infrared; SH, second harmonic.

In addition to SPDC, spontaneous four-wave mixing (SFWM) also offers excellent performance for pair production on-chip^28^. Figure 4 compares demonstrated brightness values versus waveguide optical losses for both SPDC and SFWM. To compare the usable photon pairs for applications, we considered the maximum reported photon-pair generation rates in the literature. All the input power levels required are readily achievable with integrated laser sources^29^. Although the photogalvanic-induced *χ*^(2)^ nonlinearity in our system is weak, the combination of a high quality factor and high spectral coherence ensures that the generated photons are tightly centred within a narrow bandwidth leading to high brightness. Preservation of the fidelities and interference visibilities of the quantum states is facilitated during transport by chip integration. Ultra-low-loss waveguides could improve these metrics over chip-scale distances, as well as over greater distances, such as for introducing a time delay.

In contrasting SFWM with SPDC, it can be seen that SPDC requires a near-visible pump light source. However, in the present Si_3_N_4_ system, the photogalvanic effect can efficiently generate the SPDC pump as the second harmonic of a telecom pump^16^. As a result, the entire integrated SPDC system (including pair source and pump) can operate from a single telecom semiconductor laser pump in a manner like that employed for SFWM. This concept is illustrated in the inset of Fig. 4. Furthermore, SPDC provides excellent pump and photon-pair isolation (octave-span) because of the intrinsic octave-span nature of the process. Even though the octave-span separation of photon pairs from the pump wave is possible in SFWM^30^, phase-matching requires that the signal and idler photons are widely separated in wavelength. Besides the non-equivalent optical loss in transporting such widely separated photon pairs across a chip, integrated components typically feature very different characteristics over such wide optical spans. As a result, the ability of SPDC to generate photon pairs that are both well isolated from their pump yet still within the telecom band is a possible advantage.

In summary, we have demonstrated a strong SPDC process and verified its quantum nature in a non-centrosymmetric material. Moreover, the material, Si_3_N_4_, is the dominant photonic material for ultra-low-loss integration with other photonic devices, including actives such as III–V semiconductor lasers^18,19^. The high *Q* factors give rise to the concentrated frequency distribution and spectral separability of the down-converted photon pairs, which are important for interactions with atomic and narrow-linewidth solid-state systems in real applications. The ability to integrate both pump lasers (780 nm) and near-infrared lasers (1,560 nm) with the SPDC cavity also provides a way to refresh the space charge to ensure that the system can work repetitively. Integration with other components will also enable more complex systems on-a-chip that rely upon the SPDC process. Overall, the addition of the SPDC process to the suite of capabilities provided by Si_3_N_4_ offers significant opportunities for the photonic integration of quantum systems^31,32^.

## Methods

### Effective *χ*^(2)^ creation through SHG

**Extended Data Fig. 1:**
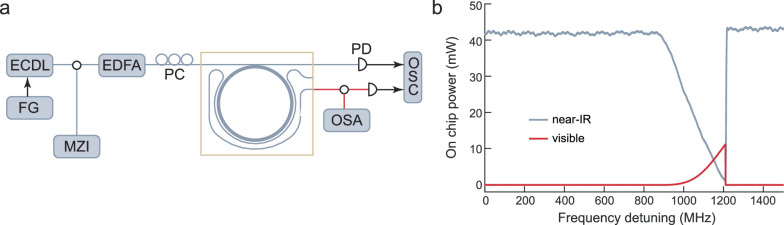
Experimental setup and characterization of second harmonic generation. **a** An external cavity diode laser (ECDL) is amplified by an erbium-doped fiber amplifier (EDFA), and then coupled to the resonator chip using a lensed fiber. The chip-waveguide is designed to couple power in the near-IR (1560 nm) to the high-*Q* silicon nitride microresonator. The SH harmonic signal is coupled to the second waveguide. A microwave function generator (FG) enables frequency sweeping of the laser, and the frequency sweep is measured using a calibrated Mach-Zehnder interferometer (MZI). The transmitted 1560 nm signal and the generated SH signal are collected and sent into two photodetectors (PDs), separately. The detected electrical signals are sent into an oscilloscope (OSC). Also shown are PC: polarization controller, OSA: optical spectrum analyzer. **b** After the space-charge grating builds up, the on-chip transmitted pump power (blue) and generated SH power (red) are recorded when scanning the frequency of the pump laser across a cavity resonance.

To induce an effective *χ*^(2)^ and simultaneously quasi-phase-match the down-conversion process, a space-charge grating was created using the experimental set-up shown in Extended Data Fig. 1a. The temperature of the microresonator was carefully controlled to frequency-match the visible mode and the near-infrared cavity mode. These frequencies were monitored using a near-infrared band tunable laser (1,560 nm) that was frequency-doubled using a periodically poled lithium niobate crystal. Once this was done, the frequency of the near-infrared laser was tuned to the cavity resonance. A periodic space-charge field then built up and generated second-harmonic power, as described elsewhere^16^. A steady state was achieved within a few seconds, and the grating persisted for a long time without the external excitation. The second-harmonic power conversion efficiency and *χ*^(2)^ strength were then characterized using a lower-power near-infrared pump power, as shown in Extended Data Fig. 1b. We observed an 11-mW on-chip 780 nm second-harmonic signal when 41 mW of 1,560 nm laser power was launched into the waveguide. This corresponds to a second-harmonic efficiency (*η*) of 651% per watt. We believe this value is limited by saturation of the second-harmonic process and the presence of cascaded sum-frequency generation to 520 nm (ref. ^42^).

For comparison, the second-harmonic efficiency can also be estimated from the measured photon-pair generation rate *R* and visible (780 nm) pump power *P*_vis_ in the SPDC process^10^:

1 $$R=\frac{{\kappa }_{{\rm{IR,L}}}^{3}}{8{\kappa }_{{\rm{IR,}}{\rm{e}}}^{2}}\eta {P}_{{\rm{vis}}},$$

where *κ*_IR,L_ (*κ*_IR,e_) is the loaded (external) cavity loss rate of the near-infrared mode. At 1.5-mW on-chip pump power, the photon-pair generation rate was measured as 800 kHz. Using the above expression and optical loss rates from the main text, the calculated SHG efficiency *η* = 624% per watt, in close agreement with the above measured value. As an aside, we expect that this calculation underestimated the peak SHG efficiency, because, as noted in the main text, the charge distribution faded away in the presence of the 780 nm pump (see Methods for more details). In particular, the 800 kHz rate was recorded a few minutes after the 780 nm pump had been coupled into the resonator, and the charge distribution would, therefore, not be as strong as the initial state. Overall, the agreement of these two inferred efficiency values might be fortuitous in view of the experimental uncertainties.

### SPDC spectral measurements

We used a a low-noise liquid-nitrogen-cooled spectrometer for the spectral measurements (PyLoN IR 1024-1.7). It had a quantum efficiency of more than 75%. The spectrometer had a 300 lines per millimetre grating (1.2 μm blaze) and an efficiency of more than 50% for wavelengths below 1,600 nm.

### Temperature dependence of the photon-pair wavelengths

**Extended Data Fig. 2:**
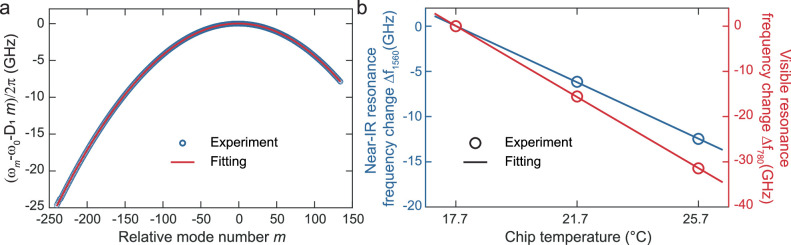
Measurement of resonator dispersion relation and resonance temperature tuning coefficients. **a** Measured frequency dispersion (blue circles) of the mode family in the near-IR band is plotted versus the relative mode number *m*. The solid red curve is a parabolic fit using *D*_1_/2π = 35.09 GHz and *D*_2_/2π = −863.7 kHz. In the plot, the mode frequencies are offset by the linear term in the Taylor expansion to make clear the second-order group dispersion, and *ω*_0_ is chosen so that *m* = 0 frequency matches to the the pump mode in the SHG process (1557.372 nm). **b** Measured frequency tuning of both near-IR (left axis) and visible (right axis) cavity mode frequencies as a function of temperature. The frequencies of the cavity mode resonances at 17.7 °C are chosen as a reference, separately.

The wavelengths of the generated photon pairs are determined by the frequency-matching condition between the visible pump mode and the near-infrared-band mode family. To determine this matching condition, the dispersion of the near-infrared mode family was first measured using a calibrated Mach–Zehnder interferometer in combination with a wavelength-tunable laser^43^. The resonant frequency *ω*_*m*_ of mode *m* can be approximated as a Taylor series relative to a mode at *ω*_0_ defined to have a relative mode number *m* = 0:

2 $${\omega }_{m}={\omega }_{0}+{D}_{1}m+\frac{1}{2}{D}_{2}{m}^{2}+\sum _{j > 2}\frac{1}{j!}{D}_{j}{m}^{j},$$

where *D*_*j*_ is the *j*th-order dispersion coefficient. Specifically, *D*_1_/2π equals the FSR, and *D*_2_ is the second-order dispersion coefficient at mode *m* = 0. As shown in Extended Data Fig. 2a, a second-order expansion agrees well with the measurements over a range of mode numbers within a few hundreds of *m* = 0. The parabolic fitting gives *D*_2_/2π = −863.7 kHz.

The resonant frequency tuning coefficients *δω*/*δT* were directly measured by varying the temperature of the microresonator chip using a thermoelectric cooler. The results are shown in Extended Data Fig. 2b. Using these measurements, the relative frequency detuning rate (*δω*_vis_/*δT* − 2*δω*_IR_/*δT*) was determined to be −814.8 MHz K^−1^, where *ω*_vis_ and *ω*_IR_ represent the resonance frequency of the near-visible and near-IR modes respectively. Combined with the negative *D*_2_ measured above, the temperature detuning coefficient changed the SPDC process from degenerate to non-degenerate on changing the resonator temperature. Accordingly, suppose that the degenerate SPDC process is frequency-matched to near-infrared mode number *m* = 0 at temperature *T*_0_ (*ω*_vis_(*T*_0_) = 2*ω*_0_(*T*_0_)). Then, using the dispersion expansion (equation (2)), the non-degenerate frequency-matching condition at temperature *T* can be written as,

3 $${\omega }_{{\rm{vis}}}(T)={\omega }_{m}(T)+{\omega }_{-m}(T)=2{\omega }_{0}(T)+{D}_{2}{m}^{2},$$

4 $${D}_{2}{m}^{2}={\omega }_{{\rm{vis}}}(T)-2{\omega }_{0}(T)=(\delta {\omega }_{{\rm{vis}}}/\delta T-2\delta {\omega }_{{\rm{IR}}}/\delta T)(T-{T}_{0}).$$

where mode numbers  +*m* and  −*m* are the relative mode numbers of the near-infrared modes involved in the SPDC process. From equation (4) and using the measured value for *δω*_vis_/*δT* − 2*δω*_IR_/*δT*, the quadratic coefficient of the chip temperature as a function of the photon-pair wavelength was calculated to be 0.0131 K nm^−^^2^, which was used to plot the quadratic function in Fig. 2d.

### Bandwidth of the down-converted photon pairs

In the spontaneous down-conversion process, a few near-infrared mode pairs can frequency-match with the visible pump mode because a slight frequency mismatch caused by dispersion can be smaller than the resonator linewidth. The frequency-matching condition can be written as:

5 $${D}_{2}{m}_{\max }^{2}-{D}_{2}{m}_{\min }^{2}=2\Delta \omega ,$$

where  ±*m*_max_ (±*m*_min_) is the largest (smallest) relative mode number of the frequency-matched mode pairs, and Δ*ω* = *ω*/*Q* is the full-width at half-maximum of the near-infrared resonances. In the degenerate case, *m*_min_ = 0. *m*_max_ is then given by,

6 $${D}_{2}{m}_{{\rm{Deg}},\max }^{2}=2\Delta \omega ,$$

Using current resonator values, *m*_Deg,__max_ was calculated to be 5.7, which is consistent with the dark blue trace in Fig. 2c.

A condition for *m*_Deg,__max_ = 1 is given by *D*_2_ > 2Δ*ω* = 2*ω*_0_/*Q* where *Q* is the loaded cavity *Q* factor of the near-infrared modes. By rewriting *D*_2_ in terms of the FSR Δ*ν*_FSR_ (hertz) and the waveguide group velocity dispersion parameter *β*_2_ (ref. ^43^), the following design condition for single-mode SPDC emerges:

7 $$Q\Delta {\nu }_{\text{FSR}}^{2} > \frac{n}{{\rm{\pi }}{{\lambda }}_{0}| {\beta }_{2}| },$$

where *λ*_0_ and *n* are the wavelength and effective index of the mode *m* = 0, respectively. *β*_2_ is related to *D*_2_ as ${\beta }_{2}=-n{D}_{2}/c{D}_{1}^{2}$, and was calculated to be 540 ps^2^ km^−1^, using the parameters from the previous section. *c* stands for the speed of light in the vacuum. For other values typical of the ultra-low-loss Si_3_N_4_ system, this gives *Q*Δ*ν*_FSR_^2^ > 5.49 × 10^11^ GHz^2^. Assuming *Q* = 100 million, which is readily attainable by this system, Δ*ν*_FSR_ > 74 GHz is sufficient to guarantee single-mode degenerate SPDC.

In the non-degenerate case, the equation can be modified as:

8 $$2\Delta \omega =2{D}_{2}\overline{m}\Delta m,$$

where $\overline{m}=({m}_{\text{max}}+{m}_{\min })/2$ and $\Delta m=({m}_{\text{max}}-{m}_{\min })/2$. At 23.7 °C, $\overline{m}=16$ and Δ*m* was calculated to be 1. This is again consistent with observations, as for both long and short wavelengths of the spectrum, photon fluxes were observed in only one neighbouring mode on each side of the strongest mode. At even higher temperatures, no side peaks were observed in the spectrum.

### *g*^(2)^ measurements

The *g*^(2)^(*t*) and pair generation rates were measured with a pair of 1.55-μm SNSPDs with a quantum efficiency of 85% provided by ID Quantique. The signals from the SNSPDs were recorded by a ID900 time-to-digital converter with a temporal resolution of 2 ns.

### Temporal degradation of the effective *χ*^(2)^

As mentioned in the main text, the SPDC rate was observed to decay in time. We believe that this results from multiphoton excitation of conduction band electrons by the 780 nm pump. These electrons would gradually neutralize the space charge, thereby diminishing the effective *χ*^(2)^. This section investigates the speed of this effect. It is experimentally challenging to reconstruct the decay directly from the SPDC rate, because the SPDC rate is sensitive to temperature and laser detuning. Alternatively, we chose to monitor the charge through the SHG signal when scanning the 1,560 nm laser across the resonance. We recorded the peak value of the second-harmonic signal, as shown in Extended Data Fig. 1b. This measurement samples a range of different detunings and should be less affected by the temperature change.

**Extended Data Fig. 3:**
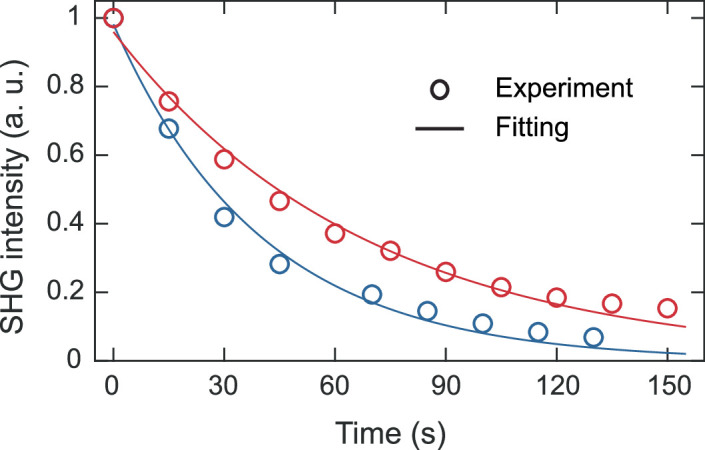
Measured *χ*^(2)^ intensity decay as a function of time at different visible pump powers. The second-harmonic intensity is normalized to the initial condition after writing of the space-charge grating. The red (blue) circles refer to the on-chip pump power of 0.5 (1.5) mW and are fitted by a exponential function (solid lines). The decay lifetime is fitted to be 68 (40) s, respectively.

The experimental set-up is the same as in Extended Data Fig. 1a. The space-charge grating was first introduced through SHG as described in the section ‘Effective *χ*^(2)^ creation through SHG’. Then, the 1,560 nm laser was turned off and the 780 nm laser was tuned to resonance to simulate the condition for SPDC. After each 15-s interval, the 780 nm signal was turned off and the 1,560 nm laser was scanned across the resonance to collect the second-harmonic peak value. Two on-chip 780 nm pump powers (0.5 and 1.5 mW) were used. The result is shown in Extended Data Fig. 3. An exponential decay fitted to the data gives lifetimes of 68 and 40 s for pumping powers 0.5 and 1.5 mW, respectively. As expected, the decay was faster with the higher pump power. Also note that as time increased, the data significantly deviated from the exponential fit and the decay rate decreased. Possible mechanisms that could impact the SHG efficiency include changes in the pump laser phase or polarization. As the SPDC measurement in the main text requires a wait time of a few minutes for the chip temperature to achieve a steady state, the observed decay times are consistent with our observation that the SPDC rate can be observed over several minutes.

## Online content

Any methods, additional references, Nature Portfolio reporting summaries, source data, extended data, supplementary information, acknowledgements, peer review information; details of author contributions and competing interests; and statements of data and code availability are available at 10.1038/s41586-025-08662-3.

## Data Availability

The data are clearly represented in Figs. 1–4 and Extended Data Figs. 1–3.

## Code availability

The code to produce the plots is available from the corresponding author (such as for comparison with a new platform).
